# Facing facts about deliberate practice

**DOI:** 10.3389/fpsyg.2014.00751

**Published:** 2014-07-17

**Authors:** David Z. Hambrick, Erik M. Altmann, Frederick L. Oswald, Elizabeth J. Meinz, Fernand Gobet

**Affiliations:** ^1^Department of Psychology, Michigan State UniversityEast Lansing, MI, USA; ^2^Department of Psychology, Rice UniversityHouston, TX, USA; ^3^Department of Psychology, Southern Illinois University EdwardsvilleEdwardsville, IL, USA; ^4^Institute of Psychology, Health, and Society, University of LiverpoolLiverpool, UK

**Keywords:** deliberate practice, music, expertise, expert performance, individual differences, talent

More than 20 years ago, Ericsson and colleagues proposed that “individual differences in ultimate performance can largely be accounted for by differential amounts of past and current levels of practice” (Ericsson et al., 1993, p. 392). We empirically tested this claim through a meta-analysis of studies of music and chess (Hambrick et al., 2014). The claim was not supported. Deliberate practice accounted for about one-third of the reliable variance in performance in each domain, leaving most of the variance explainable by other factors.

Focusing on music, Platz et al. (2014) identified 13 studies of the relationship between deliberate practice and performance and found a correlation of 0.61 after correcting for unreliability. We credit Platz et al. for their effort and thank them for their criticisms of our meta-analysis. However, none of these criticisms challenge our conclusion that deliberate practice is not as important as Ericsson and colleagues have argued.

Platz et al.'s (2014) major criticism targets our conclusion that deliberate practice accounted for 30% of the variance in music performance. They write that “relationships between variables should be interpreted in terms of linear relationships” (p. 10), and that “it is incorrect to interpret our findings (*r_c_* = 0.61) as evidence that DP explains 36% of the variance in attained music performance” (p. 11). They base this criticism on Hunter and Schmidt's (2004) argument that effect sizes from meta-analyses (and primary research) be reported as correlations rather than estimates of variance accounted for (i.e., *r*s rather than *r*^2^s).

Platz et al.'s (2014) criticism is puzzling for two reasons. First, other researchers have characterized the importance of deliberate practice in terms of variance (individual differences) accounted for—including not only Ericsson et al. (1993), but also two authors of the Platz et al. article (Reinhard Kopiez and Andreas Lehmann). For example, Kopiez and colleagues concluded that “the total life practice time at the beginning of the study correlated moderately with the baseline performance values and predicted *only 17% of their variance*” (Jabusch et al., 2009, p. 80, italics added; see also Lehmann and Ericsson, 1996; Kopiez and Lee, 2006, 2008). Second, Hunter and Schmidt's (2004) point is not that *r*^2^ is statistically incorrect. Indeed, *r* and *r*^2^ are both standard indexes of effect size (Cohen, 1988), providing different ways to conceptualize the strength of statistical relationships. Rather, their point is that *r*^2^ can make theoretically and practically important relationships seem trivially small—as when a correlation of, say, 0.30 between a predictor and an outcome is dismissed because “only” 9% of the variance is explained. For this reason, we reported both *r* and *r*^2^ values in our meta-analysis. Moreover, to avoid trivializing the role of deliberate practice, we have repeatedly emphasized its importance—the necessity of it for becoming an expert. In no less a public forum than the opinion pages of *The New York Times*, two of us commented that there is no denying the “power of practice” (Hambrick and Meinz, 2011). Again, our conclusion is not that deliberate practice is unimportant, either statistically or theoretically; it is that deliberate practice is *not as important as Ericsson and colleagues have argued*, in the precise sense that factors other than deliberate practice account for most of the variance in performance. Platz et al. apparently miss this point.

Platz et al. (2014) also take aim at the criteria we used for including a study in our meta-analysis, calling them “intuitive” (p. 4). In fact, our criteria were dictated by the theoretical claim we sought to test and were clearly stated in our article—measures of accumulated amount of deliberate practice and performance were collected and a correlation between these measures was reported. Platz et al. did find a few studies in their literature search that we did not, but this does not bear on our conclusion that deliberate practice is not as important as Ericsson and colleagues have argued. In fact, the results of Platz et al.'s meta-analysis support this conclusion: A correlation of 0.61 between deliberate practice and music performance leaves room for two additional orthogonal predictors of nearly the same magnitude (*r*s = 0.56).

Perhaps with an inkling of this, Platz et al. (2014) argue that their correlation of 0.61 might be regarded as the “theoretically lower bound of the true effect of DP” (p. 11) because “time estimations of practice durations are only approximate indicators of deliberate practice” (p. 11). But their correlation could equally well be regarded as an *upper* bound on the true effect of deliberate practice. For example, using retrospective questionnaires to measure deliberate practice could lead to inflated correlations between deliberate practice and performance if people base practice estimates on their skill rather than recollections of engaging in practice. The more general problem with Platz et al.'s argument is that it can always be made: if the correlation between deliberate practice and performance is not as high as one likes, one can always argue that this is because the measure of deliberate practice is imperfect—making it impossible to falsify hypotheses about the predictive value of deliberate practice.

Finally, some measures used by Platz et al. (2014) may not be estimates of deliberate practice. For example, for some studies, they used the correlation between number of accompanying performances and sight-reading performance, but number of accompanying performances could be considered a measure of what Ericsson et al. (1993) termed “work,” as distinct from deliberate practice. Platz et al. are also inconsistent in what they consider the accumulation period for deliberate practice (e.g., lifetime for some studies, to age 18 for others).

The bottom line is that, in all major domains in which deliberate practice has been studied, most of the variance in performance is explained by factors other than deliberate practice (Macnamara et al., 2014). These factors may include starting age (Gobet and Campitelli, 2007), working memory capacity (Meinz and Hambrick, 2010), and genes (Hambrick and Tucker-Drob, 2014). For scientists, the task now is to develop and test falsifiable theories of expertise that include as many relevant constructs as possible.

## Conflict of interest statement

The authors declare that the research was conducted in the absence of any commercial or financial relationships that could be construed as a potential conflict of interest.
